# Apoptosis, Mitosis, and Cell Turnover in Oral Potentially Malignant Disorders and Oral Squamous Cell Carcinoma: A Narrative Review on Histochemical Assessment and Clinicopathological Relevance

**DOI:** 10.7759/cureus.115951

**Published:** 2026-09-08

**Authors:** Swati S Gotmare, Treville Pereira, Sanpreet S Sachdev, Subraj Shetty

**Affiliations:** 1 Oral Pathology and Microbiology, DY Patil University School of Dentistry, Navi Mumbai, IND; 2 Oral Pathology and Microbiology, Bharati Vidyapeeth (Deemed to be University) Dental College and Hospital, Navi Mumbai, IND

**Keywords:** acridine orange, feulgen reaction, methyl green-pyronin, nuclear morphometry, oral epithelial dysplasia

## Abstract

Maintenance of oral epithelial integrity depends on the equilibrium between cellular proliferation, differentiation, and programmed cell death. Disruption of this equilibrium contributes to the development and progression of oral potentially malignant disorders (OPMDs) and oral squamous cell carcinoma (OSCC). Apoptotic index (AI), mitotic index (MI), turnover index (TI), and nuclear-cytoplasmic morphometric parameters provide measurable representations of epithelial kinetics and cytological atypia. This narrative review examines alterations in these parameters across oral leukoplakia, oral epithelial dysplasia (OED), oral submucous fibrosis (OSMF), and OSCC. It also evaluates hematoxylin and eosin (H&E), methyl green-pyronin Y, Feulgen, fluorescent Feulgen, and acridine orange staining for identifying apoptotic bodies, mitotic figures, and nuclear alterations. Available evidence indicates that apoptotic and proliferative activity generally increases with epithelial dysplasia and malignancy, although the relationship is not consistently linear. Histochemical techniques can enhance nuclear contrast and facilitate quantitative assessment, particularly in settings where immunohistochemistry or molecular testing is unavailable. Nevertheless, variation in fixation, staining, field selection, counting criteria, and observer calibration limits comparison across studies. Moreover, associations with histological grade should not be interpreted as evidence of prognostic prediction without longitudinal clinical outcomes. Standardized counting protocols, appropriate normal controls, blinded observer assessment, digital image analysis, and validation against established proliferation and apoptosis markers are required. Quantitative cell-turnover assessment should presently be regarded as an adjunct to conventional histopathological diagnosis rather than a replacement for established grading systems.

## Introduction and background

The oral epithelium maintains its structural and functional integrity through a regulated balance among cellular proliferation, differentiation, and programmed cell death. Disruption of this balance may permit genetically altered cells to survive and expand, thereby contributing to the development of oral potentially malignant disorders (OPMDs) and oral squamous cell carcinoma (OSCC). OPMDs comprise a clinically diverse group of conditions associated with an increased risk of oral cancer, among which oral leukoplakia and oral submucous fibrosis (OSMF) are particularly important in populations with prevalent tobacco and areca-nut use [1].

Evasion of apoptosis and sustained proliferative signaling are closely related features of oral carcinogenesis. Alterations in cell-cycle regulators, apoptosis-related proteins, DNA-repair mechanisms, and intracellular signaling pathways have been documented during the transformation of normal oral epithelium into dysplasia and invasive malignancy [2]. At the tissue level, these alterations may be represented by the apoptotic index (AI), mitotic index (MI), turnover index (TI), and nuclear-cytoplasmic morphometric parameters. Studies of oral epithelial dysplasia (OED) and OSCC have generally demonstrated higher apoptotic and mitotic activity with increasing histological severity, although the relationship is not consistently linear and considerable overlap exists between diagnostic groups [3,4].

Routine hematoxylin and eosin (H&E) examination remains the diagnostic standard; however, apoptotic bodies and mitotic figures may be difficult to distinguish from pyknotic nuclei, inflammatory cells, and processing artifacts. Histochemical stains such as methyl green-pyronin Y, Feulgen, fluorescent Feulgen, and acridine orange differentially enhance DNA and/or RNA contrast, making condensed chromatin, fragmented nuclei, and mitotic chromosomes more conspicuous than in routine H&E-stained sections [4,5]. These techniques may therefore facilitate the identification and quantitative assessment of apoptotic, mitotic, and nuclear alterations.

Nevertheless, the available evidence remains fragmented across different oral lesions, histochemical techniques, and quantitative parameters. Differences in staining protocols, tissue compartments, field-selection methods, counting denominators, and definitions of AI, MI, and TI limit comparison among studies. Furthermore, associations between these indices and histological grade have sometimes been interpreted as indicating prognostic value despite the limited availability of longitudinal clinical outcomes. A consolidated assessment of their biological meaning, methodological limitations, and clinicopathological relevance is therefore required.

In this context, the present narrative review examines alterations in apoptosis, mitosis, epithelial turnover, and nuclear morphology across oral leukoplakia, OED, OSMF, and OSCC. It also compares the principal histochemical techniques used for their assessment, identifies important methodological sources of variability, and critically evaluates the diagnostic, grading, and prognostic relevance of the available evidence.

## Review

Narrative review framework

A structured narrative approach was used to bring together biological, histopathological, histochemical, and clinical evidence. This manuscript was prepared according to the six domains of the Scale for the Assessment of Narrative Review Articles (SANRA): importance of the review, statement of its aims, description of the literature search, appropriate referencing, scientific reasoning, and presentation of relevant evidence [6].

Information sources and search strategy

PubMed/MEDLINE, Embase, Scopus, and Google Scholar were searched from database inception to August 9, 2026. The reference lists of relevant original studies and review articles were also examined to identify additional publications. The search included terms related to OPMDs, OSCC, apoptosis, mitosis, epithelial turnover, nuclear morphometry, histochemical staining, and image analysis. The complete search strategy for each database is provided in the Appendices.

Scope and selection of literature

Human studies involving tissue sections or oral cytological specimens from normal oral mucosa, oral leukoplakia, OED, OSMF, or OSCC formed the main literature considered in this review. Emphasis was placed on studies evaluating apoptotic bodies, mitotic figures, AI, MI, TI, nuclear or cellular dimensions, nuclear-cytoplasmic relationships, and relevant staining methods. Immunohistochemical, molecular, and digital-pathology studies were also considered when they provided useful biological or methodological information.

Animal-only investigations, nonoral lesions, isolated case reports, and publications without information relevant to epithelial kinetics, nuclear morphology, or the staining methods under consideration fell outside the scope of the review. Original studies were prioritized for clinical and histopathological observations, while reviews, consensus reports, and foundational cytochemical publications were used to explain the biological and technical background. Sources were selected according to their relevance to the aims of the narrative review.

Organization of evidence and narrative synthesis

Formal study-level data extraction was not undertaken because this was a narrative review. The literature was organized according to the biological process, staining technique, clinicopathological category, quantitative parameter, and proposed clinical use. Comparisons focused on the general direction and consistency of the findings, differences in staining and counting methods, and limitations affecting interpretation. Statistical pooling was not performed because the studies differed in their diagnostic classifications, staining protocols, tissue compartments, field-selection methods, counting denominators, and outcome definitions.

Oral epithelial homeostasis and altered cell kinetics

Apoptosis is an energy-dependent form of regulated cell death characterized by cell shrinkage, chromatin condensation, nuclear fragmentation, membrane blebbing, and the formation of apoptotic bodies [7]. In normal stratified squamous epithelium, cellular proliferation is mainly confined to the basal and parabasal layers. Differentiating cells migrate toward the surface and are eventually shed.

Carcinogenesis affects both cellular proliferation and cell death. Oncogenic signaling promotes continued cellular division, while defective apoptotic pathways allow cells with genomic damage to survive. Sustained proliferative signaling and resistance to cell death are therefore closely related features of cancer [8]. Their histological features include expansion of the proliferative compartment, suprabasal mitoses, nuclear enlargement, hyperchromatism, cellular pleomorphism, abnormal mitotic figures, and altered apoptotic activity.

An increase in visible apoptosis does not necessarily indicate regression. Rapidly proliferating neoplastic tissue may also show considerable cell death because of genomic instability, hypoxia, nutrient deprivation, immune-mediated injury, and failed mitosis. AI and MI should therefore be interpreted together and within their tissue context.

Morphological recognition of apoptosis and mitosis

Apoptotic cells usually show rounded cellular shrinkage, dense chromatin condensation, nuclear fragmentation, and small membrane-bound apoptotic bodies. These structures may be rapidly removed by neighboring cells or macrophages without producing the marked inflammation associated with necrosis [9]. Pyknotic nuclei, inflammatory cells, keratohyalin granules, necrotic debris, tangentially sectioned nuclei, and tissue-processing artifacts may resemble apoptotic bodies.

Mitotic figures lack an intact nuclear membrane and show condensed chromosomes arranged in prophase, metaphase, anaphase, or telophase. Abnormal forms include multipolar, asymmetrical, lag-type, and ring mitoses. Such forms reflect disturbed chromosomal segregation and have been described during oral tumorigenesis [10]. In addition, folded tissue, crushed cells, apoptotic nuclei, lymphocytes, and nonmitotic hyperchromatic nuclei may be mistaken for mitotic figures. Clear morphological criteria, adequate tissue preservation, and observer training are therefore needed for reliable identification.

Histochemical methods

Hematoxylin and Eosin Staining

H&E staining remains the reference method for diagnosis, assessment of tissue architecture, and histological grading. Its advantages include wide availability, low cost, familiarity, and compatibility with routine light microscopy. However, H&E is not specific for apoptosis or mitosis. Small apoptotic bodies and mitotic figures may be obscured by hyperchromatic nuclei, inflammation, necrosis, or processing artifacts. Comparative studies suggest that special stains can improve nuclear contrast and make apoptotic and mitotic structures easier to recognize [4,5,11]. Representative apoptotic and mitotic cells in H&E-stained sections are shown in Figure 1.

**Figure 1:**
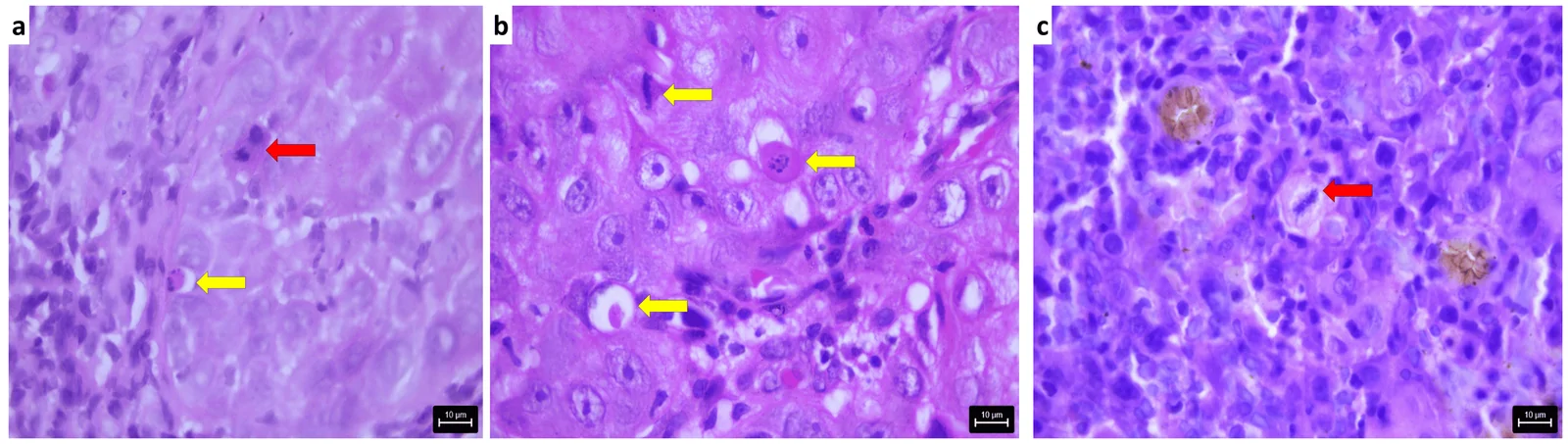
Photomicrograph of hematoxylin and eosin-stained sections under 100X magnification showing (a) an apoptotic cell (yellow arrow) and another cell undergoing mitosis (red arrow), (b) apoptotic cells (yellow arrows), and (c) a mitotic cell in metaphase (red arrow) Image courtesy: Dr. Swati Gotmare

Feulgen Staining

The Feulgen reaction is a classical DNA-specific technique used for the demonstration and quantitative assessment of nuclear DNA [12]. Controlled acid hydrolysis removes purine bases and exposes aldehyde groups in deoxyribose. These groups react with Schiff reagent to produce magenta nuclear staining. When the procedure is carefully controlled, staining intensity is proportional to DNA content [12,13].

Feulgen staining provides clear nuclear definition and has been used to identify mitotic figures and assess nuclear morphology in OED and OSCC. Comparative studies have reported better visualization of the chromosomal material and less confusion between mitotic figures and other hyperchromatic structures [11,14]. Feulgen-based morphometry has also shown increases in nuclear area, perimeter, diameter, and nuclear-cytoplasmic ratio with poorer OSCC differentiation [15]. Representative mitotic phases demonstrated by Feulgen staining are shown in Figure 2.

**Figure 2:**
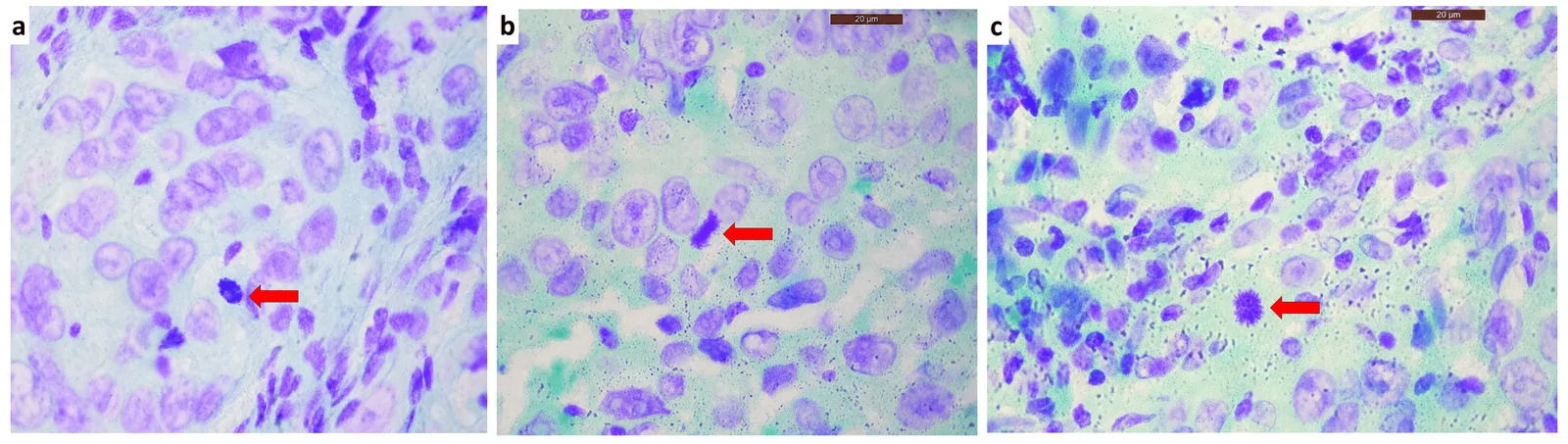
Photomicrograph showing mitotic cells in (a) anaphase, (b) metaphase, and (c) prophase (Feulgen stain, 100X) Image courtesy: Dr. Swati Gotmare

The main limitations of the Feulgen reaction are its sensitivity to fixation, hydrolysis time, section thickness, and tissue age. Inadequate hydrolysis produces weak staining, whereas excessive hydrolysis may reduce the staining signal. These factors must be standardized when the technique is used for nuclear morphometry or quantitative DNA assessment.

Methyl Green-Pyronin Y Staining

Methyl green-pyronin Y is a differential nucleic-acid stain. Foundational studies showed that methyl green preferentially stains highly polymerized DNA, while pyronin has a greater affinity for lower nucleic-acid polymers and RNA-rich structures [16]. Methyl green therefore stains DNA green or blue-green, while pyronin Y demonstrates RNA in red or reddish-purple shades.

The contrasting colors may make condensed chromatin, apoptotic fragments, nucleoli, and cytoplasmic RNA more visible. Methyl green-pyronin Y has been used to identify apoptotic bodies, mitotic figures, nucleoproteins, and nuclear alterations in oral leukoplakia, OED, OSMF, and OSCC [4,5,17]. Representative apoptotic cells demonstrated by this stain are shown in Figure 3.

**Figure 3:**
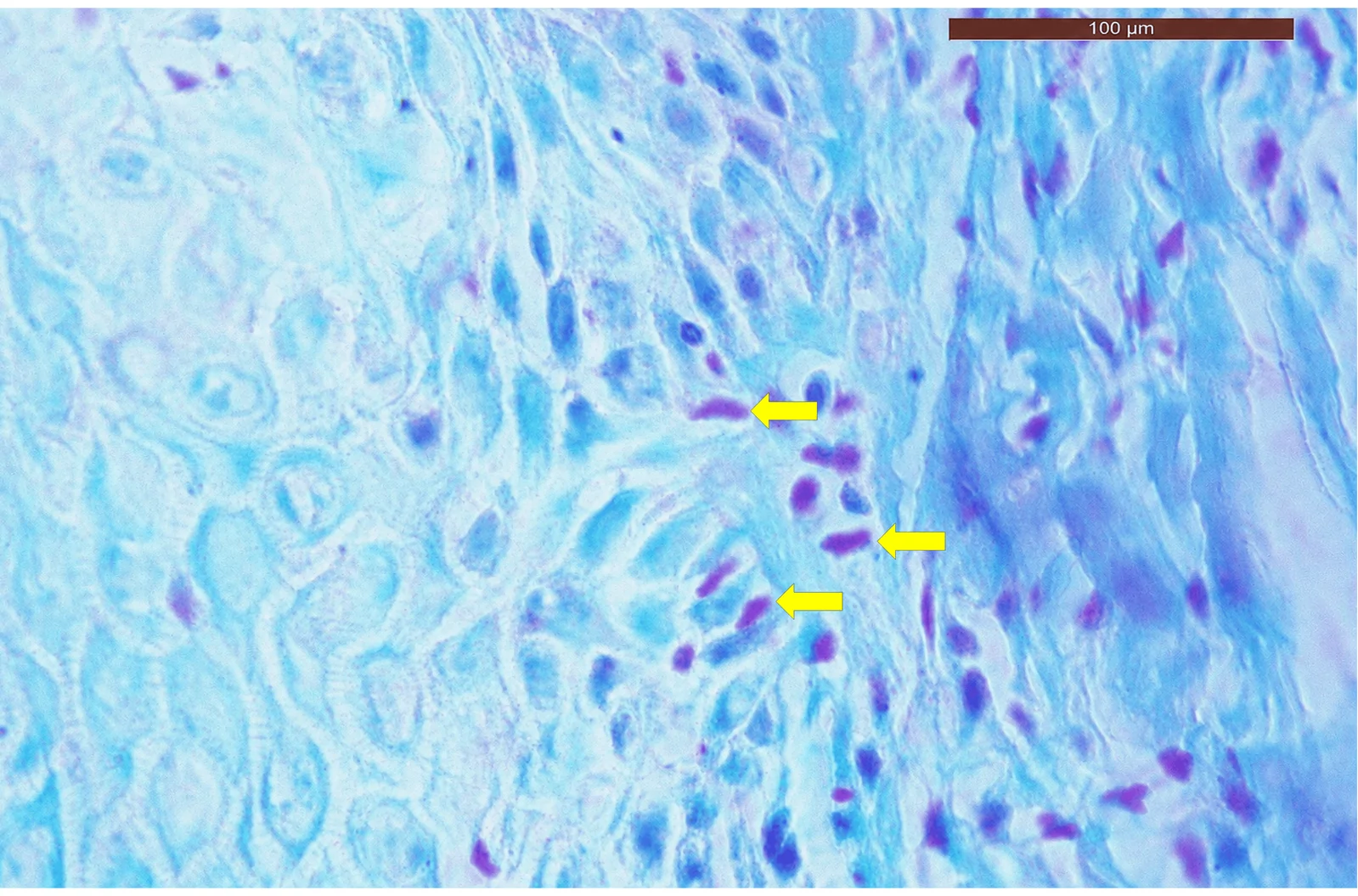
Photomicrograph showing apoptotic cells (arrows) (methyl green-pyronin Y stain under 100X magnification) Image courtesy: Dr. Swati Gotmare

Nucleomorphometric studies using methyl green-pyronin Y have reported changes in nuclear size, cellular dimensions, and nuclear-cytoplasmic relationships across OED, OSMF, and OSCC [18,19]. However, the stain is not specific for apoptosis or malignant transformation. Fixation, dye purity, solution preparation, pH, differentiation, and staining duration may affect the final color. Standardized procedures and suitable controls are therefore necessary.

Fluorescent Feulgen Staining

Fluorescent Feulgen techniques use a fluorescent Schiff-type reagent after controlled acid hydrolysis. Fluorescence may provide high contrast between DNA-containing structures and the surrounding tissue, making condensed chromosomes, fragmented nuclei, and nuclear boundaries easier to identify [13,20].

Possible advantages include better visualization of small nuclear structures and compatibility with digital image acquisition. However, evidence validating fluorescent Feulgen techniques in oral lesions remains limited. Background autofluorescence, filter selection, exposure time, reagent stability, fading, and slide-storage conditions may affect interpretation. The technique should therefore be considered investigational until it is directly compared with conventional Feulgen and H&E staining in well-designed studies.

Acridine Orange Staining

Acridine orange is a metachromatic fluorescent stain that interacts with nucleic acids. Double-stranded DNA generally produces green fluorescence, while RNA and single-stranded nucleic acids may show orange-red fluorescence depending on the concentration, pH, and staining conditions. Oral cytological studies have reported rapid staining and improved visualization of nuclear abnormalities using acridine orange [21,22].

The technique may help identify nuclear enlargement, irregular nuclear outlines, increased nucleic-acid content, apoptotic fragments, and mitotic activity. However, acridine orange is not specific for malignancy or apoptosis. Reactive cells, inflammatory cells, and microorganisms may also show intense fluorescence. The stained slides are prone to fading and usually require prompt examination or controlled digital imaging.

The main features of the histochemical techniques are compared in Table 1.

**Table 1:** Comparison of the principal histochemical staining methods H&E: hematoxylin and eosin

Staining method	Principal target	Major advantage	Major limitation	Suggested application
H&E	General nuclear and cytoplasmic morphology	Demonstrates complete tissue architecture and remains the reference stain for diagnosis and grading [4,5]	Apoptotic bodies and mitotic figures may resemble pyknotic nuclei or cellular debris [4,5,11]	Primary diagnosis and comparison with special stains
Conventional Feulgen	DNA exposed through controlled acid hydrolysis and Schiff reaction	Provides clear nuclear contrast and allows DNA-associated morphometry under controlled conditions [12,13]	Requires careful control of fixation, hydrolysis, section thickness, and staining time	Identification of mitotic figures, nuclear morphometry, and quantitative DNA assessment [11,14,15]
Methyl green-pyronin Y	DNA and RNA demonstrated in contrasting colors	May improve visualization of apoptotic, mitotic, and nuclear structures [4,5,16,17]	Protocol-dependent and not specific for apoptosis or malignancy	Assessment of apoptotic bodies, mitotic figures, nucleoproteins, and nuclear morphology [17-19]
Fluorescent Feulgen	DNA demonstrated using a fluorescent Schiff-type reagent	Produces high nuclear contrast and may support digital image analysis [13,20]	Limited validation in oral lesions, autofluorescence, and fading	Research-based assessment of nuclear structures and DNA-associated changes
Acridine orange	DNA and RNA demonstrated by different fluorescence patterns	Allows rapid visualization of nucleic acids and nuclear abnormalities [21,22]	Reactive and inflammatory cells may fluoresce, and preparations are prone to fading	Adjunctive oral cytology and fluorescence-based assessment of nuclear changes [20-22]

Quantitative indices of epithelial turnover

Histomorphometric studies commonly use AI, MI, TI, the apoptotic-to-mitotic ratio, and nuclear-cytoplasmic measurements to assess epithelial kinetics. Their calculations, interpretations, and main limitations are summarized in Table 2.

**Table 2:** Quantitative parameters used for epithelial cell-kinetics assessment AI: apoptotic index; MI: mitotic index; TI: turnover index

Parameter	Recommended calculation	Biological interpretation	Principal limitation
AI	Number of apoptotic cells or bodies ÷ total epithelial or tumor cells counted × 100 [3-5,11]	Proportion of visible apoptotic cells within the examined tissue	Apoptotic bodies are short-lived and may be confused with pyknotic nuclei, inflammatory cells, or debris
MI	Number of mitotic figures ÷ total epithelial or tumor cells counted × 100 [3,11,14,15]	Proportion of cells visibly undergoing mitosis	Represents only the short mitotic phase and is strongly affected by field selection
TI	AI + MI [3,23,24]	Combined visible apoptotic and proliferative activity	Does not directly measure net tissue growth or show whether proliferation exceeds cell loss
Apoptotic-to-mitotic ratio	AI ÷ MI [3,23,24]	Relationship between visible cell death and cellular division	Becomes unstable when MI is very low or zero; validated clinical thresholds are unavailable
Nuclear-cytoplasmic ratio	Nuclear area ÷ cytoplasmic area [15,18,19]	Relative nuclear enlargement and reduced cytoplasmic maturation	Some studies divide nuclear area by total cell area, resulting in noncomparable values
Nuclear area	Area enclosed by the nuclear boundary [15,18,19]	Reflects nuclear enlargement and cytological atypia	Sectioning plane, overlapping cells, and segmentation errors may alter measurements
Cell area	Area enclosed by the cellular boundary [18,19]	Reflects cellular dimensions and cytoplasmic maturation	Cellular boundaries may be unclear, particularly in tissue sections

TI represents the sum of visible apoptotic and mitotic activity. It should not be interpreted as a direct measurement of tumor growth because tissues with identical TI values may have different relationships between cellular proliferation and cell loss. The apoptotic-to-mitotic ratio should also be interpreted carefully when very few mitotic figures are present.

Cell turnover across the clinicopathological spectrum

Normal Oral Mucosa

Normal oral mucosa generally shows low AI and MI, with mitotic activity concentrated in the basal and parabasal layers. Mature superficial cells have relatively larger cytoplasmic areas and smaller nuclei. Normal tissue is an important comparator because staining intensity and absolute counts may vary between laboratories. Studies without a normal-control group may identify differences among lesional grades but cannot determine the degree of deviation from normal epithelial turnover.

Oral Leukoplakia and Oral Epithelial Dysplasia

Oral leukoplakia is a clinical diagnosis with variable histopathological appearances, ranging from hyperkeratosis without dysplasia to severe OED. Histological dysplasia remains the most important tissue-based indicator of malignant potential. However, grading is subjective, and only some dysplastic lesions progress to malignancy.

Progression from mild to severe OED is commonly associated with the expansion of the proliferative compartment, increased mitotic activity, suprabasal mitoses, abnormal mitotic figures, reduced cellular maturation, and nuclear enlargement. AI may also increase as dysplastic cells accumulate genomic damage. Morphological studies have generally reported greater apoptotic and mitotic activity in OED than in normal mucosa, with further increases in OSCC [3,4,23,24]. Nevertheless, overlap between grades prevents the use of an isolated index as a reliable diagnostic cutoff or predictor of malignant transformation.

Oral Submucous Fibrosis

OSMF is a chronic and progressive OPMD strongly associated with areca-nut exposure. A systematic review estimated an overall malignant-transformation proportion of approximately 4%, although the contributing studies have demonstrated substantial heterogeneity and risk of bias [25]. Several clinical, functional, and histopathological systems have been proposed for classifying or staging OSMF [26].

The connective-tissue changes include progressive collagen deposition, hyalinization, reduced vascularity, and muscular degeneration. The epithelium may show atrophy, hyperkeratosis, or dysplasia. Altered expression of p53, Ki-67, Bcl-2, and Bax suggests disturbed proliferative and apoptotic regulation [27]. Increased Ki-67 expression and expansion of the proliferative compartment have also been reported with progressive disease [28,29].

The connective-tissue stage of OSMF must be distinguished from the presence and severity of epithelial dysplasia. Advanced fibrosis does not necessarily indicate severe epithelial atypia. Epithelial AI and MI should therefore be correlated separately with OSMF stage and OED grade.

Oral Squamous Cell Carcinoma

OSCC shows loss of epithelial architecture, invasion, nuclear pleomorphism, abnormal mitotic activity, and variable apoptosis. Poorly differentiated tumors may have greater proliferative activity. However, mitotic and apoptotic counts can differ within the same tumor because of keratinization, necrosis, hypoxia, inflammation, and clonal heterogeneity.

Morphological studies have generally reported higher AI and MI in OSCC than in dysplastic epithelium [3,23,24]. In one study, tumors without regional lymph-node involvement showed more apoptotic bodies than tumors with nodal involvement, whereas MI did not differ significantly [30]. Such findings are potentially important but require confirmation in larger longitudinal studies.

Nuclear-cytoplasmic morphometry

Nuclear enlargement and relative cytoplasmic reduction are established features of epithelial dysplasia and malignancy. Measured parameters include nuclear area, nuclear perimeter, minimum and maximum nuclear diameter, cell area, cytoplasmic area, nuclear-cytoplasmic ratio, and nuclear circularity [15,18,19].

An increasing nuclear-cytoplasmic ratio has been reported across OPMDs and OSCC. However, comparison between studies is difficult because different formulas are used. Nuclear area divided by total cell area is not equivalent to nuclear area divided by cytoplasmic area, where:

Cytoplasmic area = total cell area − nuclear area

The formula, segmentation method, calibration scale, magnification, and handling of overlapping cells should be reported clearly. Measurements from tissue sections and exfoliative cytology should also be interpreted separately because their cellular boundaries and processing artifacts differ.

Comparison with immunohistochemical markers

Histochemical indices demonstrate visible morphological events but provide limited information about the underlying molecular pathway. Ki-67 identifies cells in active phases of the cell cycle but does not specifically demonstrate mitosis. Phosphohistone H3 is more closely restricted to late G2 and mitosis and may provide a more reproducible estimate of mitotic activity than morphology alone [31]. Minichromosome maintenance protein 2 identifies replication-licensed cells and may demonstrate a wider proliferative compartment than Ki-67 [32].

Apoptosis may be evaluated using cleaved caspase-3, terminal deoxynucleotidyl transferase dUTP nick-end labeling, Bax, Bcl-2, and related markers. These methods provide molecular information but also have limitations. DNA-fragmentation assays may label nonapoptotic cellular injury, while apoptosis may occur without detectable activation of a particular caspase [2].

Special stains should not be presented as replacements for immunohistochemistry or molecular testing. Their main advantages are affordability, preservation of morphology, rapid use, and possible application as quantitative adjuncts in laboratories with limited resources.

Methodological sources of variability

OED grading is affected by interobserver and intraobserver variability [33]. Observer-calibration exercises may improve agreement, showing the importance of standard definitions and training before quantitative assessment [34]. The main methodological sources of variability are summarized in Table 3.

**Table 3:** Methodological sources of variability and recommended approaches

Methodological component	Potential source of variability or bias	Recommended approach
Fixation and tissue processing	Delayed, inadequate, or prolonged fixation may alter nuclear morphology and staining intensity [13,15,17]	Standardize fixation and document tissue-processing conditions
Section thickness	Inconsistent thickness may produce overlapping nuclei or fragmented nuclear structures	Maintain the same section thickness across specimens
Field selection	Hotspot selection may increase counts, while random fields may miss focal activity [3,11,14,15]	Define whether fields are selected randomly, systematically, or from hotspots and use the same method across groups
Counting denominator	Small or inconsistent cell or field counts produce unstable indices [3,14,15]	Use a fixed denominator and report it clearly
Tissue compartment	Basal, parabasal, suprabasal, superficial, invasive, and perinecrotic regions differ in biological activity [3,23,27-29]	Define the compartment before counting and analyze biologically distinct regions separately
Histopathological grading	Grading is affected by observer variability [33,34]	Use accepted criteria and conduct observer-calibration exercises
Observer assessment	Small apoptotic bodies and mitotic figures may be interpreted subjectively [3-5,9,11]	Use clear criteria and, where possible, blinded and calibrated observers
Image calibration and segmentation	Incorrect thresholding or boundary detection may affect nuclear and cellular measurements [15,18,19]	Report the software, magnification, calibration, segmentation, and manual-correction methods
Staining quality	Reagent preparation, hydrolysis, pH, staining time, and fading may affect visualization [4,5,12,13,16,20-22]	Standardize staining and include suitable procedural controls
Outcome interpretation	Cross-sectional associations with grade may be incorrectly described as prognostic [23-25,30]	Reserve prognostic claims for longitudinal studies evaluating transformation, recurrence, metastasis, or survival

Diagnostic relevance

Diagnostic discrimination refers to differentiation among normal mucosa, OPMDs, and OSCC. AI, MI, TI, and nuclear morphometric parameters may support this distinction. However, overlap between diagnostic groups prevents their independent use as diagnostic criteria. These measurements should be interpreted together with clinical findings, conventional histopathology, and accepted grading criteria.

Relevance to histological grading

Several cross-sectional studies have reported associations between quantitative indices and increasing OED severity or decreasing OSCC differentiation. However, histological grading is observer-dependent, and differences in staining, field selection, tissue compartment, and counting methods limit the development of reliable grade-specific thresholds. An index associated with a more severe grade should not automatically be considered a measure of disease progression.

Prognostic relevance

Prognostic value requires evidence that an index predicts malignant transformation, nodal metastasis, recurrence, treatment response, or survival. Cross-sectional differences between histological grades cannot establish such prediction. AI, MI, TI, and morphometric parameters should therefore be described as associated with histological grade rather than predictive of future clinical outcomes. Longitudinal studies are needed to determine whether they provide information beyond age, lesion site, clinical appearance, exposure history, OED grade, tumor stage, and treatment.

Digital pathology and artificial intelligence

Digital image analysis may reduce observer fatigue and provide continuous measurements of nuclear size, shape, staining intensity, and spatial distribution. Applications of artificial intelligence in OED assessment are increasing, although limited external validation, explainability, and generalizability remain important concerns [35].

Recent convolutional neural network models have shown promising results in differentiating normal epithelium and different grades of OED [36]. However, high accuracy within an internal image dataset does not prove clinical validity. Future models should use whole-slide images from different laboratories and scanners and should be tested against meaningful clinical outcomes. Apoptotic index and MI features could be combined with architectural dysplasia criteria. Such systems should assist the pathologist and allow manual confirmation of apoptotic bodies, mitotic figures, and nuclear boundaries.

Implications for resource-constrained settings

Methyl green-pyronin Y, conventional Feulgen, and acridine orange are relatively inexpensive and may be used where immunohistochemistry or molecular testing is limited. Their main uses may include teaching, quantitative research, observer training, and preliminary assessment of epithelial kinetics.

However, low reagent cost does not by itself establish clinical usefulness. Laboratories require standardized preparation, suitable controls, appropriate microscopes, observer training, and reproducibility testing. Fluorescence-based techniques also require stable light sources, suitable filters, controlled exposure, and timely slide examination.

Limitations of the current evidence

Most available studies are single-center and cross-sectional, with relatively small samples. Diagnostic groups, grading systems, staining protocols, field-selection procedures, counting denominators, and tissue compartments vary considerably. Normal controls are not consistently included, and many studies do not report observer agreement.

Apoptotic bodies are rapidly removed and may be underestimated in static tissue sections. MI captures only the short morphological phase of mitosis. TI has no accepted clinical cutoff and does not show whether proliferation exceeds cell loss. Evidence validating fluorescent Feulgen staining in oral lesions also remains limited.

Few studies have linked histochemical indices with future malignant transformation, recurrence, metastasis, or survival. These limitations prevent firm conclusions regarding prognostic value and restrict the development of clinically useful thresholds.

Limitations of the review

This review has limitations related to its narrative design. Sources were selected according to their relevance to the review objectives rather than through formal independent screening and study-level data extraction, and no standardized risk-of-bias tool was applied. Although four databases and the reference lists of relevant publications were searched, some relevant evidence may have been overlooked. These limitations were considered by using cautious language and avoiding quantitative or prognostic conclusions that were not supported by the available evidence.

Future directions

Future studies should include adequately sampled normal oral mucosa, nondysplastic OPMDs, different grades of OED, OSMF with and without epithelial dysplasia, and different grades of OSCC. Fixation, section thickness, staining procedures, field selection, tissue compartment, and counting criteria should be standardized. AI, MI, TI, and the apoptotic-to-mitotic ratio should be reported separately because TI alone cannot show whether cellular proliferation exceeds cell loss.

Studies evaluating nuclear-cytoplasmic relationships should state the formula used and describe image calibration and segmentation. Morphological measurements should be assessed by blinded and calibrated observers, with interobserver and intraobserver agreement reported. Histochemical findings should also be compared with established markers such as Ki-67, phosphohistone H3, cleaved caspase-3, and terminal deoxynucleotidyl transferase dUTP nick-end labeling.

Multicenter longitudinal studies are needed to determine whether these indices independently predict malignant transformation, nodal metastasis, recurrence, or survival. Digital image-analysis and artificial-intelligence models should use whole-slide images from different institutions and should undergo external validation before clinical use.

## Conclusions

Alterations in apoptosis, mitosis, epithelial turnover, and nuclear morphology are commonly observed across OED, OSMF, and OSCC, although their relationship with disease severity is not always consistent. H&E remains the diagnostic standard, while methyl green-pyronin Y, Feulgen, fluorescent Feulgen, and acridine orange may improve nuclear visualization and support quantitative assessment. These techniques should presently be considered adjuncts to conventional histopathology rather than independent diagnostic or prognostic tools. Standardized methods and longitudinal studies are needed before these indices can be used reliably for clinical risk assessment.

## Appendices

**Table 4:** Database-specific search strategies

Database	Search strategy	Search period
PubMed/MEDLINE	(("oral potentially malignant disorder*"[Title/Abstract] OR "oral leukoplakia"[Title/Abstract] OR "oral epithelial dysplasia"[Title/Abstract] OR "oral submucous fibrosis"[Title/Abstract] OR "oral precancer*"[Title/Abstract] OR "oral squamous cell carcinoma"[Title/Abstract]) AND (apoptosis[Title/Abstract] OR "apoptotic index"[Title/Abstract] OR mitosis[Title/Abstract] OR "mitotic index"[Title/Abstract] OR "turnover index"[Title/Abstract] OR "cell proliferation"[Title/Abstract] OR "nuclear cytoplasmic ratio"[Title/Abstract] OR "nuclear morphometry"[Title/Abstract]) AND ("hematoxylin and eosin"[Title/Abstract] OR "methyl green pyronin"[Title/Abstract] OR Feulgen[Title/Abstract] OR "fluorescent Feulgen"[Title/Abstract] OR "acridine orange"[Title/Abstract] OR "fluorescence microscopy"[Title/Abstract] OR histochemistry[Title/Abstract] OR "image analysis"[Title/Abstract]))	Database inception to August 9, 2026
Embase	('oral potentially malignant disorder*':ti,ab OR 'oral leukoplakia':ti,ab OR 'oral epithelial dysplasia':ti,ab OR 'oral submucous fibrosis':ti,ab OR 'oral precancer*':ti,ab OR 'oral squamous cell carcinoma':ti,ab) AND ('apoptosis':ti,ab OR 'apoptotic index':ti,ab OR 'mitosis':ti,ab OR 'mitotic index':ti,ab OR 'turnover index':ti,ab OR 'cell proliferation':ti,ab OR 'nuclear cytoplasmic ratio':ti,ab OR 'nuclear morphometry':ti,ab) AND ('hematoxylin and eosin':ti,ab OR 'methyl green pyronin':ti,ab OR 'feulgen':ti,ab OR 'fluorescent feulgen':ti,ab OR 'acridine orange':ti,ab OR 'fluorescence microscopy':ti,ab OR 'histochemistry':ti,ab OR 'image analysis':ti,ab)	Database inception to August 9, 2026
Scopus	TITLE-ABS-KEY("oral potentially malignant disorder*" OR "oral leukoplakia" OR "oral epithelial dysplasia" OR "oral submucous fibrosis" OR "oral precancer*" OR "oral squamous cell carcinoma") AND TITLE-ABS-KEY(apoptosis OR "apoptotic index" OR mitosis OR "mitotic index" OR "turnover index" OR "cell proliferation" OR "nuclear cytoplasmic ratio" OR "nuclear morphometry") AND TITLE-ABS-KEY("hematoxylin and eosin" OR "methyl green pyronin" OR Feulgen OR "fluorescent Feulgen" OR "acridine orange" OR "fluorescence microscopy" OR histochemistry OR "image analysis")	Database inception to August 9, 2026
Google Scholar	("oral potentially malignant disorder" OR "oral leukoplakia" OR "oral epithelial dysplasia" OR "oral submucous fibrosis" OR "oral squamous cell carcinoma") ("apoptotic index" OR "mitotic index" OR "turnover index" OR "nuclear morphometry" OR "cell proliferation") (Feulgen OR "methyl green pyronin" OR "acridine orange" OR histochemistry OR "image analysis")	Database inception to August 9, 2026
